# When the Day Goes Awry: Families Caring for Persons With Dementia

**DOI:** 10.1093/geroni/igaa057.2190

**Published:** 2020-12-16

**Authors:** Karen Roberto, Jyoti Savla, Steven Zarit

## Abstract

The daily lives of family caregivers of persons with dementia (PwD) often require that they manage multiple competing demands in a context of unpredictability. Memory and behavior changes associated with dementia can cause PwD to act in random and irrational ways that create stress and influence all aspects of caregivers’ everyday life. Supportive others, including informal helpers and formal service professionals, should provide relief to primary caregivers; however, help may not alleviate caregiver stress and can sometimes compound the burden of care. This symposium draws on daily diary surveys and face-to-face interviews to focus on four aspects of managing everyday care of PwD among family caregivers in rural areas. Brandy Renee McCann explores how caregivers’ vigilance on behalf of PwD care quality interacts with service use. Karen Roberto examines the ways in which caregivers manage PwD resistance to help, including their use of forceful care strategies. Rosemary Blieszner focuses on competing caregiver roles and demands that may contribute to or alleviate caregiver stress. Tina Savla addresses the unexpected, and often hidden, challenges involved in using formal services. Collectively, the four presentations provide in-depth insight into the complicated daily lives of families coping with dementia and the ways in which they meet the demands of full-time caregiving under often difficult and challenging circumstances. Discussant Steve Zarit considers the efficacy of these management strategies for various aspects of everyday care and offers suggestions for future research and person-centered programs and interventions to reduce health disparities among caregivers in rural areas.

